# The Effects of Sleeve Gastrectomy on Blood Pressure, Blood Pressure Variability, and Autonomic Functions in Severely Obese Patients Without Diabetes or Hypertension

**DOI:** 10.3390/jcm15051820

**Published:** 2026-02-27

**Authors:** Metin Karayakalı, Zeki Özsoy

**Affiliations:** 1 Department of Cardiology, Tokat Gaziosmanpaşa University, Tokat 60250, Turkey; 2 Department of General Surgery, Tokat Private Medical Park Hospital, Tokat 60230, Turkey

**Keywords:** laparoscopic sleeve gastrectomy, ambulatory blood pressure monitoring, blood pressure variability, heart rate variability, heart rate turbulence

## Abstract

**Background/Objectives:** Laparoscopic sleeve gastrectomy (LSG) treats severe obesity, but data on its effects on 24 h blood pressure (BP) patterns, blood pressure variability (BPV), and cardiac autonomic nervous system (CANS) in obese patients without hypertension or diabetes are limited. We evaluated these parameters before and after LSG. **Methods:** 78 patients with severe obesity (BMI ≥ 40 kg/m^2^) without hypertension or diabetes who underwent LSG between January 2016 and December 2019 were included in the study. Patients underwent ambulatory blood pressure monitoring (ABPM), ambulatory electrocardiographic monitoring, and laboratory tests before and six months after surgery. **Results:** Preoperative ABPM was characterized by a significant proportion of masked hypertension (43.5%), high 24 h BP (mean SBP 138.9 ± 5.5 mmHg, DBP 81.1 ± 4.9 mmHg), high BP load (39% SBP, 38% DBP), and a non-dipper pattern (67.9%). After LSG, significant improvements were observed in mean 24 h SBP, DBP (*p* < 0.001), BPV, BP load, and non-dipper patterns. HRV parameters (SDANN, RMSSD) increased significantly (*p* < 0.001) and HRT parameters improved: TO became more negative from −0.54 ± 1.73 to −2.53 ± 1.97, TS increased from 5.98 ± 3.49 to 9.87 ± 4.28 ms/RR (*p* < 0.001). We found a strong association between decreased body mass index and BP changes. Changes in glucose, HbA1c, and HOMA-IR predicted CANS improvement (β = 0.24–0.38; R^2^ = 20.8–29.7%). **Conclusions:** Six months after LSG, significant improvements in BP, BPV, and CANS were observed. BP reduction was primarily associated with weight loss, while glucose control was associated with autonomic improvements. LSG was associated with early improvements in surrogate cardiovascular risk markers through combined weight-dependent and metabolic-hormonal mechanisms.

## 1. Introduction

According to current estimates, approximately 880 million adults are living with obesity. A comparison of data from 1975 and 2022 revealed a three-fold increase in obesity prevalence among females and a four-fold increase among males, resulting in nearly 504 million females and 374 million males living with obesity [1]. Severe obesity, according to recent United States data, affects 9.4% of the population [2]. The gradual increase in obesity is associated with an increase in associated health risks, particularly in cardiovascular mortality and disease rates. Hypertension (HT), diabetes mellitus (DM), arrhythmia, cardiomyopathies, heart failure, and coronary artery disease are the main conditions that are at high risk of developing due to obesity [3].

Obesity-related HT results from the additive effects of several factors, including obstructive sleep apnea, increased sympathetic nervous system and renin–angiotensin–aldosterone system activity, insulin resistance, endothelial dysfunction, and chronic inflammation. Visceral adipose tissue accumulation increases the release of adipokines (leptin, adiponectin), cytokines (TNF-α, IL-6), and free fatty acids, triggering insulin resistance and vascular inflammation. This pathophysiological cascade not only increases HT prevalence in obese individuals but also accelerates target organ damage and major adverse cardiovascular events [4].

Bariatric surgery is considered the gold standard for the treatment of severe obesity, and among different surgical techniques, laparoscopic sleeve gastrectomy (LSG) is one of the two most commonly performed procedures due to its low complication rates and effective weight loss [5]. The GATEWAY (Gastric Bypass to Treat Obese Patients With Steady Hypertension) study demonstrated superiority of bariatric surgery over medical therapy in hypertensive obese patients [6]. This study showed that in the surgical group, 80.7% of patients reduced antihypertensive medications, and 46.9% achieved HT remission (compared with 13.7% and 2.4%, respectively, in the medical therapy group) [6]. However, the effects of LSG in severely obese patients without diagnosed HT or DM but with high-normal blood pressure masked HT identified through ambulatory blood pressure monitoring (ABPM) remain inadequately elucidated. This patient group has cardiovascular risk due to masked HT and non-dipping blood pressure pattern, which cannot be detected by standard office blood pressure measurements in the preoperative period.

ABPM has a stronger predictive value for cardiovascular endpoints compared to office blood pressure measurements. By measuring blood pressure at regular intervals over 24 h, ABPM allows for the assessment of diurnal variations, nocturnal blood pressure profiles, and the white coat effect, thus more accurately reflecting the true blood pressure burden on patients [7]. Through ABPM, significant prognostic parameters such as blood pressure variability (BPV), blood pressure load (BPL), and nocturnal blood pressure dipping can be evaluated [8]. BPV is associated with arterial stiffness and predicts target organ damage independent of mean blood pressure [9]. Blood pressure variability is increased in obese individuals and has been associated with endothelial dysfunction, oxidative stress, and vascular inflammation [10]. Blood pressure load represents an independent predictor of left ventricular hypertrophy and cardiovascular events [11]. A nocturnal blood pressure decrease of 10–20% relative to daytime values is defined as a normal dipping pattern and indicates cardiovascular health but non-dipping or reverse dipping is associated with increased cardiovascular risk [12]. Changes in BPV, BPL, and dipping pattern after LSG are an important indicator for understanding the full spectrum of cardiovascular benefits provided by the surgical procedure.

About 15% of people with normal clinic blood pressure actually have masked hypertension [13]. Masked hypertension prevalence is higher in younger individuals and those with obesity, diabetes, CKD, family history of hypertension, or high-normal office BP [13]. Meta-analyses and recent studies demonstrate that masked hypertension carries cardiovascular risk comparable to sustained hypertension and substantially higher than normotension.

Cardiac autonomic dysfunction is known to be associated with obesity. Obesity-related autonomic disorder is characterized by increased sympathetic activity and decreased parasympathetic activity [14]. Heart rate turbulence (HRT) and heart rate variability (HRV) provide non-invasive assessment of cardiac sympathetic and parasympathetic balance and function. They are strong indicators of cardiovascular mortality risk [15]. HRV essentially measures temporal changes between consecutive heartbeats. Based on these measurements, it measures the flexibility of the heart’s response to external factors such as stress, excitement, fear, physical activity, rest and sleep [16]. This flexibility is mainly determined by changes in sympathetic and parasympathetic activity and their reciprocal balance. HRV essentially comprises time domain parameters based on the statistics of the temporal change in the R-R intervals and frequency domain parameters based on the frequency of fluctuations in heart rate [16]. Reduced HRV indicates cardiac autonomic imbalance and increases sudden cardiac death risk [17]. HRT describes heart rate changes following ventricular premature beats; Turbulence Onset (TO) and Turbulence Slope (TS) parameters serve as indicators of baroreflex sensitivity [18]. In obese patients, HRV is reduced and HRT is impaired; however, limited data exist regarding whether improvement in these parameters occurs following LSG and which factors (weight loss, glycemic control, insulin sensitivity) are associated with such improvement [19].

The effects of LSG on blood pressure and the autonomic nervous system cannot be explained solely by weight loss. The rapid hormonal and neurohumoral effects of surgery should also be taken into account. Prouroguanylin (ProUGN)/uroguanylin increases after surgery and provides natriuresis and diuresis by inhibiting NHE3 in the proximal tubule [20]. Cholecystokinin (CCK) levels rise and suppress renal sympathetic activity, downregulating intrarenal RAAS [21]. Incretin gut hormones such as GLP-1 and peptide YY increase, which balances sympathetic tone and promotes diuresis and natriuresis [22]. Leptin levels rapidly decrease (within 10 days), reducing sympathetic overactivity [23]. Angiotensin II, renin, aldosterone, and endothelin-1 levels decline [24]. Neprilysin activity decreases, prolonging the half-life of vasodilator peptides (ANP, BNP) [24]. Inflammatory markers (hs-CRP, leukocytes) decrease, and endothelial function improves [25]. Renal sinus adipose tissue diminishes, and renal compression with abnormal sodium retention normalizes [26]. Together, these mechanisms may partly explain the rapid and partly weight-independent blood pressure effects observed after LSG.

The aim of this study was to evaluate the effects of LSG on BPV, HRV parameters, and HRT parameters at 6 months after surgery in severely obese patients who were not diagnosed with HT or DM but had normal, high normal blood pressure, or masked HT according to ABPM data. Furthermore, this study aims to identify important determinants of post-LSG improvements using multivariate regression analyses. Particularly, we aim to investigate the improvement in blood pressure parameters after LSG, the recovery in the cardiac autonomic nervous system as measured by HRV and HRT, and the relationship of these improved parameters with the amount of weight loss, BMI reduction, or metabolic parameters such as glucose, insulin, C-peptide, HbA1c, and HOMA-IR.

## 2. Material and Methods

### 2.1. Study Design and Patient Selection

**Figure 1:**
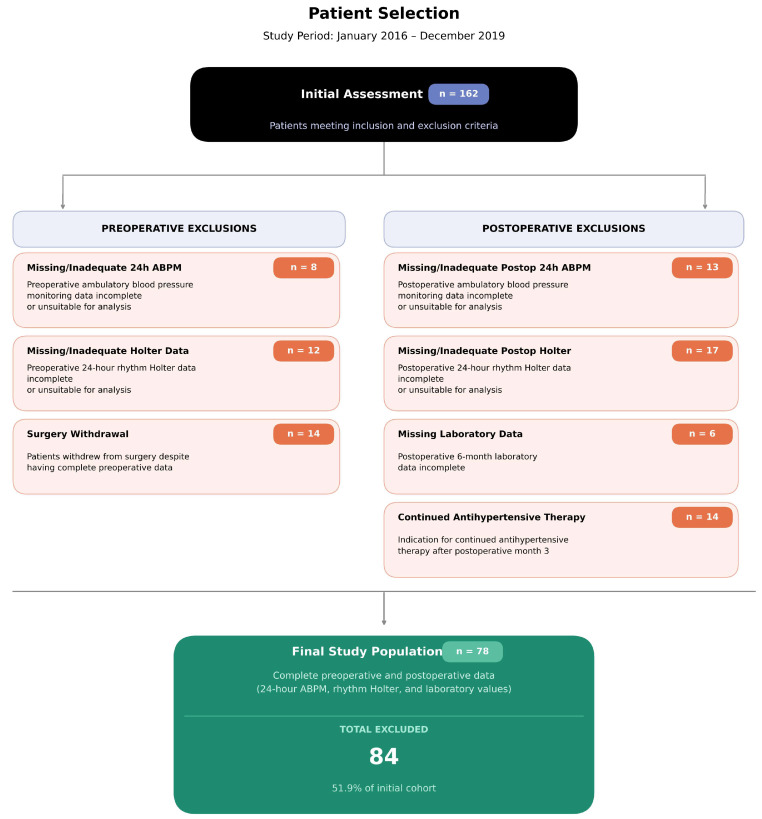
Patient selection flowchart. Study period: January 2016–December 2019. A total of 162 patients underwent initial assessment; 84 patients were excluded due to missing or inadequate data, leaving 78 patients in the final analysis. ABPM, ambulatory blood pressure monitoring.

We identified 162 patients who were evaluated for LSG at the Gaziosmanpaşa University Hospital General Surgery Clinic between January 2016 and December 2019. Of the 162 patients initially evaluated for eligibility, 84 were excluded based on predefined criteria, including missing clinical or follow-up data, inadequate ABPM or Holter recordings, or the need for prolonged antihypertensive therapy. Ultimately, 78 patients with complete and analyzable data were included in the final analysis (Figure 1). Operations were performed by the same surgical team using standard techniques. The study received ethical approval from Tokat Gaziosmanpaşa University faculty of medicine Clinical Research Ethics Committee (approval number: 15-KAEK-211, date: 15 December 2015). Written informed consent was obtained from all participants, and the study was performed according to the Helsinki Declaration principles.

The study was conducted in patients aged 18–62 years with a body mass index (BMI) ≥ 40 kg/m^2^. We excluded patients with HT, DM, arrhythmia, heart failure, significant renal or hepatic dysfunction, malignancy, or psychiatric conditions, including those on relevant medications. This study used preoperative and six-month postoperative data. Weight and height measurements were taken using identically calibrated equipment. Blood samples were collected after at least 8–12 h of fasting. Our analyses included pre- and post-operative complete blood count, total cholesterol, triglycerides, HDL-cholesterol, LDL-cholesterol, insulin, C-peptide, glucose, and HbA1c parameters as laboratory data. Transthoracic echocardiograms were performed by a single cardiologist using an Affiniti 70 device (Philips Ultrasound Inc., Bothell, WA, USA).

### 2.2. 24 h Blood Pressure Monitoring

ABPM was performed preoperatively and postoperatively using a SunTech Oscar 2 device (version 1998–2009). Blood pressure monitoring was configured with 20 min measurement intervals during daytime periods (07:00–22:00) and 30 min intervals during nighttime periods (22:00–07:00). Records with a valid evaluation rate below 75% were excluded from the study. We calculated 24 h total mean, daytime mean, and nighttime mean systolic and diastolic blood pressure, BPL (percentage of readings above 140/90 mmHg during daytime and 120/80 mmHg at night), and BPV (standard deviation and coefficient of variation). Using these data, we also calculated time-weighted systolic and diastolic blood pressure and coefficient of variability for systolic and diastolic blood pressure. These BPV parameters capture different aspects of blood pressure imbalance: the standard deviation reflects overall variability, the coefficient of variation accounts for relative changes, and the weighted standard deviation includes day-night transitions and provides complementary information about hemodynamic stability. Dipping status was defined according to the decrease in blood pressure at night. A nighttime blood pressure decrease of 10% or more was defined as dipper, and a decrease of less than 10% was defined as non-dipper. If patients were diagnosed with hypertension according to the ABPM results, lifestyle advice, salt restriction, medication, or both were applied, taking into account blood pressure values and risk factors according to current guidelines [13]. Patients who still needed to use antihypertensive medication after 3 months of pre- and post-operative follow-up were excluded from the study.

### 2.3. 24 h Holter ECG Monitoring

Holter recordings were obtained using the ELA Medical SYNESCOPE system (MultiChannel-MultiDay Version 3.0). After the obtained recordings were transferred to analysis computers, they were analyzed in terms of HRV and HRT parameters.

### 2.4. Heart Rate Variability Analysis

All participants were instructed to abstain from caffeine and stimulant foods at least 12 h prior to the measurement, and from heavy physical activity 24 h prior. Measurements were evaluated via 24 h rhythm Holter electrocardiogram (ECG) signals. In the time domain, the following parameters were calculated: standard deviation of NN intervals (SDNN), standard deviation of mean NN intervals (SDANN), root mean square of successive differences (RMSSD), percentage of successive NN intervals differing by more than 30 ms (pNN30), and percentage of successive NN intervals differing by more than 50 ms (pNN50); in the frequency domain, low frequency (LF), high frequency (HF) powers, and the LF/HF ratio representing sympathovagal balance were calculated via spectral analysis.

### 2.5. Heart Rate Turbulence

HRT is measured using the short-term fluctuations in R-R intervals following isolated VPCs. In healthy individuals, VPCs show a brief shortening (TO) followed by a gradual lengthening (TS) before returning to its baseline level [18]. Transient inhibition of vagal activity in response to ineffective ventricular contraction accounts for the early shortening, while the gradual lengthening observed thereafter is attributed to reflex vagal activation induced by rising arterial pressure mediated through the sympathetic nervous system. In conclusion, HRT is impaired in patients with reduced baroreflex [18]. Accepted normal values are <0% for TO and >2.5 ms/R-R for TS [18]. Optimal HRT calculation requires a normal sinus rhythm, the absence of other arrhythmias, and at least 5 ventricular premature contractions [18]. We excluded 25 patients (32%) from HRT analysis: 18 lacked adequate premature beat frequency, 5 had excessive atrial or ventricular arrhythmias, and 2 had recordings of insufficient quality. Ultimately, 53 patients (68%) were found to be suitable for HRT evaluation.

### 2.6. Statistical Analysis

Normally distributed continuous data were summarized using mean ± standard deviation, whereas median, minimum and maximum values were used for non-normally distributed data. The Shapiro–Wilk test determined data distribution. According to distribution normality, paired samples *t*-tests or Wilcoxon signed-rank tests compared pre- and postoperative values. Statistical relationships between categorical variables were analyzed using chi-square tests. Pearson or Spearman’s correlation tests according to distribution pattern were used for correlation analysis. Multiple linear regression analyses were used to identify important predictors of continuous variables such as BMI, weight loss, HRV and HRT parameters. Stepwise regression used entry criterion *p* < 0.05 and removal criterion *p* > 0.10. Multicollinearity was assessed using variance inflation factor (VIF < 5). Model assumptions including linearity and homoscedasticity were verified through residual diagnostics. Analyses included only patients with complete data for each variable (complete case analysis). For statistical significance and the validity of our null hypothesis, a two-tailed *p* value of less than 0.05 was accepted. Statistical analyses were performed using SPSS version 22.0 (IBM Corp., Armonk, NY, USA), and Python software (version 3.12; Python Software Foundation, Wilmington, DE, USA) was used to create some figures.

## 3. Results

Our study included 78 severely obese patients (BMI > 40 kg/m^2^) without HT or DM. The mean BMI decreased from 47 kg/m^2^ before surgery to 31 kg/m^2^ after six months; this represented a mean weight loss of 43 kg (Table 1). Insulin, C-peptide, HOMA-IR, glucose, and HbA1c decreased significantly. Total cholesterol, LDL cholesterol, and triglycerides decreased, while HDL cholesterol increased modestly (Table 1 and Table 2). While a significant decrease was examined in white blood cell and platelet counts, no significant changes were observed in blood urea nitrogen (BUN), creatinine, estimated glomerular filtration rate, potassium, and calcium levels.

**Figure 2:**
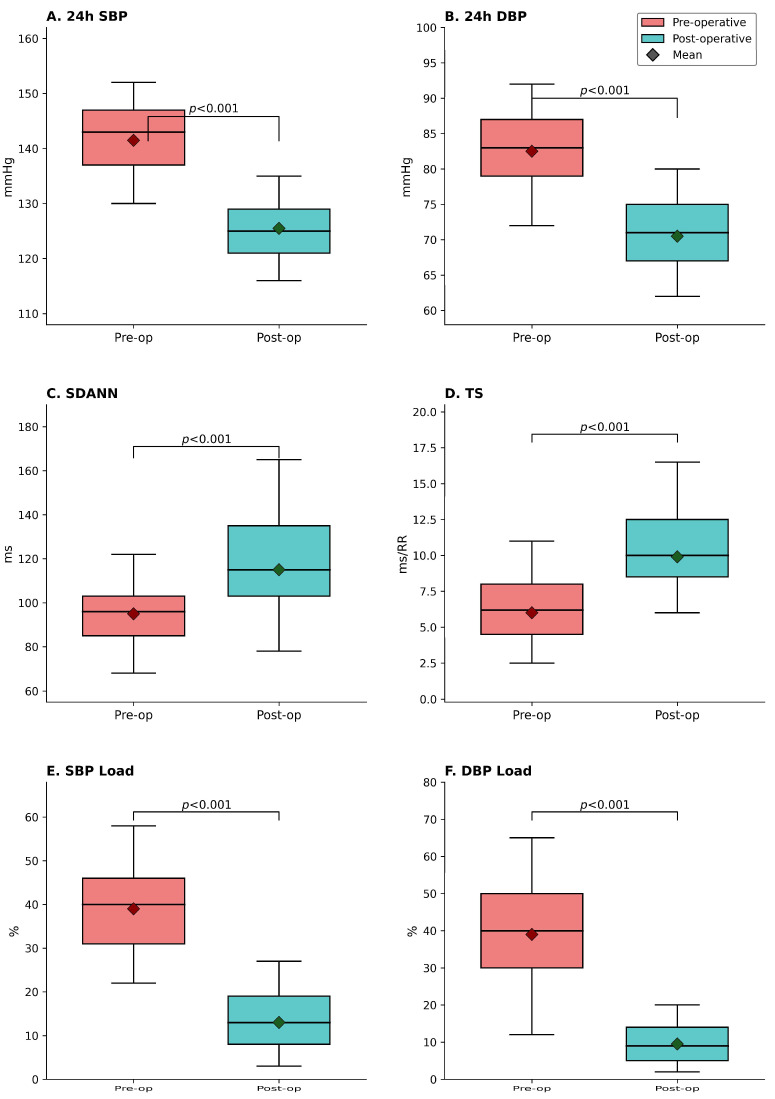
Comparison of cardiovascular and autonomic parameters before and 6 months after laparoscopic sleeve gastrectomy. (**A**) 24-h systolic blood pressure (SBP); (**B**) 24-h diastolic blood pressure (DBP); (**C**) Standard deviation of averaged NN intervals (SDANN); (**D**) Turbulence slope (TS); (**E**) SBP load; (**F**) DBP load. Box plots show median (horizontal line), mean (diamond ◆), interquartile range (box boundaries), and data range (whiskers). All comparisons showed significant improvement (*p* < 0.001). SBP, systolic blood pressure; DBP, diastolic blood pressure; SDANN, standard deviation of averaged NN intervals; TS, turbulence slope; SBP Load and DBP Load, percentage of blood pressure readings exceeding normal thresholds (daytime > 140/90 mmHg, nighttime > 120/80 mmHg).

Although the patients included in the study did not have diagnosed hypertension, preoperative ABPM measurements revealed that a significant proportion of patients met the criteria for masked hypertension. The mean 24 h SBP was 138.90 ± 5.54 mmHg and the mean 24 h DBP was 81.06 ± 4.89 mmHg. Blood pressure load was significantly high (SBP 39.17%, DBP 38.39%) and showed a non-dipper pattern in 67.9%. According to the 2024 ESC HT guideline criteria, 34 out of 78 patients (43.5%) initially met the criteria for masked hypertension [27]. Of the remaining patients, 25 (32.1%) had high normal blood pressure and 19 (24.4%) had normal blood pressure. 24 h mean systolic and diastolic pressures showed a significant decrease. Also, systolic BPL decreased from 39.17 ± 10.98% to 12.08 ± 10.05% (*p* < 0.001), and a more pronounced decrease was observed in the diastolic BPL from 38.39 ± 17.79% to 8.20 ± 9.39% (*p* < 0.001). Simultaneous significant decreases were examined in BPV parameters such as standard deviation, coefficient of variation, and weighted standard deviation. The rate of having non-dipper blood pressure decreased from 67.9% to 53.8% (*p* < 0.05) (Table 3, Figure 2).

Subgroup analysis compared patients with masked hypertension (n = 34) and those with baseline normal or high-normal blood pressure (n = 44). The masked hypertension group had higher BMI, ambulatory blood pressure values, heart rate, insulin levels, C-peptide, and HOMA-IR at baseline, whereas preoperative HRV and HRT parameters were comparable between groups. Despite these baseline differences, the magnitude of improvement in blood pressure and HRV indices after LSG did not differ significantly between groups. Greater reductions in insulin and C-peptide were observed in the masked hypertension group, whereas the change in HbA1c was similar between groups. A significant between-group difference was observed for TO change (*p* = 0.027), with a larger improvement in the normal/high-normal group (Table 4).

Table 5 and Figure 2 present HRV and HRT parameters from 24 h Holter monitoring. Mean heart rate decreased from 86.8 ± 8.6 bpm preoperatively to 76.9 ± 7.3 bpm at 6 months (*p* < 0.001). Heart rate variability parameters were low before LSG but improved at 6 months after surgery. SDANN increased from 94.7 ± 25.7 ms to 113.4 ± 31.7 ms (*p* < 0.001). RMSSD increased from 27.2 ± 10.2 ms to 36.9 ± 11.6 ms (*p* < 0.001). Similarly, significant improvements were detected in frequency domain measurements such as LF, HF, and the LF/HF ratio, as well as other time domain parameters.

Heart rate turbulence analysis was feasible in 53 patients with adequate premature ventricular contractions. Turbulence onset became more negative from −0.54 ± 1.73% to −2.53 ± 1.97% (*p* < 0.001), indicating improved baroreflex sensitivity. Turbulence slope increased from 5.98 ± 3.49 ms/RR to 9.87 ± 4.28 ms/RR (*p* < 0.001).

Correlation analyses were performed on the differences between pre-operative and 6-month post-operative values (Table 6). The decrease in BMI was correlated with improvements in mean BP and BPV. A moderate correlation was observed between weight loss and a decrease in 24 h diastolic blood pressure (r = 0.350, *p* = 0.002). Also decrease in body mass index showed a moderate correlation with a weighted standard deviation decrease in diastolic pressure (r = 0.357, *p* = 0.001). Metabolic improvements were significantly associated with BP, HRV, and HRT parameters (Table 5). Among metabolic variables, the decrease in HbA1c showed a particularly significant correlation with improvement in turbulence slope (r = 0.436, *p* = 0.001, n = 53).

**Figure 3:**
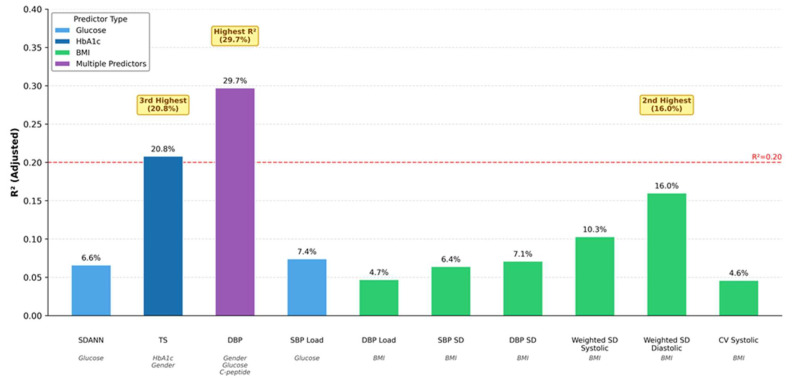
R^2^ values from multivariate regression models showing variance explained by different predictors. Higher bars indicate better model fit. Color coding represents predictor type: glucose (light blue), HbA1c (blue), BMI (green), and multiple predictors combined (purple). The highest R^2^ (29.7%) was achieved for diastolic blood pressure prediction using gender, glucose, and C-peptide. BMI, body mass index; CV, coefficient of variation; DBP, diastolic blood pressure; SBP, systolic blood pressure; SD, standard deviation; SDANN, standard deviation of averaged NN intervals; TS, turbulence slope.

Multivariate regression analyses showed that reductions in glucose, HbA1c, C-peptide, and HOMA-IR were associated with some autonomic and blood pressure outcomes. BMI reduction emerged as a significant predictor of BPV (Table 7, Figure 3). BMI reduction also predicted all blood pressure outcomes and heart rate reduction. However, metabolic parameters such as glucose, HbA1c, C-peptide, and HOMA-IR were important determinants of HRV and HRT improvements.

## 4. Discussion

In this cohort, we observed significant improvements in blood pressure profile, BPV, and cardiac autonomic function within six months after LSG in severely obese patients without HT or DM. This study provides a comprehensive evaluation of ABPM parameters, BPV, and cardiac autonomic parameters, including HRV and HRT, before and six months after LSG, and investigates factors associated with these changes. Together, these findings suggest that cardiovascular adaptation after LSG may involve different mechanisms beyond weight reduction alone, including metabolic improvement.

### 4.1. Dominant Role of Glycemic Control: Weight Loss—Significant Effect

Changes in glucose and HbA1c showed consistent associations with improvements in both autonomic and blood pressure-related parameters. In particular, improvements in glycemic indicators were associated with changes in HRV indices, mean diastolic blood pressure, systolic BPL, and baroreflex sensitivity. These findings suggest that glycemic regulation may contribute to cardiovascular and autonomic adaptation after LSG, even in patients without diabetes. Our results are consistent with some previous reports, although not all studies have reached similar conclusions. Previous work has linked HbA1c to HRV parameters in both type 1 and type 2 diabetes [28,29]. Another study showed that improvement in HRV parameters after laparoscopic Roux-en-Y gastric bypass surgery was significantly associated with HOMA-IR, and that HOMA-IR was an independent predictor of HRV improvement [30]. Together, these observations support a relationship between metabolic regulation and autonomic function. Our study suggests that glycemic control after LSG was associated with improvements in autonomic and cardiovascular benefits, even in patients without a pre-diabetic or DM diagnosis, and highlights the importance of HbA1c for autonomic function. Our study results showed that every 0.5% reduction in HbA1c was associated with significant autonomic improvement, supporting the positioning of bariatric surgery as “metabolic surgery”.

The significant reduction in LDL cholesterol observed after LSG may reflect metabolic adaptations beyond weight loss alone. Improvements in insulin sensitivity, postoperative dietary changes, and alterations in cholesterol homeostasis have been suggested as potential contributing factors [31]. These mechanisms may collectively contribute to the LDL reduction observed after bariatric surgery.

### 4.2. Effect of BMI Reduction on Blood Pressure Stability

Reduction in BMI was consistently associated with improvements in several BP and BPV parameters. Glycemic variables were not retained in most BPV models. These findings suggest that changes in BPV may be more closely related to weight reduction and hemodynamic adaptation than to glycemic parameters in this cohort.

Our findings are consistent with those reported by Kotsis et al. in obese hypertensive patients. In their study, BMI was also found to be an independent predictor of systolic and diastolic blood pressure, mean 24 h systolic blood pressure, and 24 h pulse pressure [32]. Weighted SD systolic and diastolic blood pressure data reported by Głuszewska et al., 10 days and 6 months after bariatric surgery, are consistent with our study [23]. Furthermore, subgroup analysis in that study encompassed both patients with and without HT, showing significant improvement in blood pressure parameters post-surgery [23]. However, other studies have emphasized the role of vasoactive mediators independent of BMI change [24]. A study investigated factors that prevent long-term (3 years) recurrence of hypertension and showed that the amount of weight loss was an independent predictor of hypertension recurrence [33]. Some studies in the literature have found no significant relationship between weight loss and blood pressure parameters, possibly because these studies have focused on hormones and mediators that have been proven to mediate blood pressure reduction.

### 4.3. Undiagnosed Masked Hypertension in Severe Obesity

We observed a high prevalence of masked hypertension in severely obese patients classified as normotensive according to traditional clinical criteria. Although all participants were considered normotensive in routine clinical assessment, 43.5% met the 2024 ESC criteria for masked hypertension on ABPM [27]. These results highlight the limited sensitivity of office blood pressure measurements in severe obesity. Masked HT is known to be 2–3 times more common in obesity compared to the general population, affecting 30–40% of severely obese individuals, but it largely remains undiagnosed because office measurements cannot detect it or patients do not undergo cardiological examinations for suspected HT [34]. Differences between office and ambulatory blood pressure measurements in obesity may be explained by various factors, including sleep apnea, sympathetic hyperactivity, insulin resistance, impaired sodium metabolism, and endothelial dysfunction [35].

At six months after LSG, significant improvements were observed in ambulatory blood pressure parameters in this subgroup. Mean 24 h SBP decreased by 14.5 mmHg, BPL decreased by approximately 30%, and the percentage of non-dipper pattern decreased from 68% to 54%. These findings suggest that LSG may improve ambulatory blood pressure patterns in patients with masked hypertension. These observations reinforce the clinical significance of ABPM in morbidly obese individuals. Systematic use of ABPM in the preoperative period can improve risk stratification and reveal clinically significant blood pressure phenotypes that might otherwise remain undetected.

To examine whether baseline BP phenotype influenced postoperative response, we performed a subgroup analysis comparing patients with masked hypertension to those with normal or high-normal BP. Although the masked hypertension group had higher baseline BMI, ambulatory BP, and insulin resistance, the magnitude of improvement in BP and HRV parameters after LSG was similar between groups. These findings suggest that early autonomic and ambulatory BP improvements were not substantially modified by baseline masked hypertension status. However, given the sample size, this observation should be interpreted cautiously and confirmed in larger cohorts.

### 4.4. Reduction in HOMA-IR and Insulin Resistance

HOMA-IR showed a significant association in two models. It was found to be a significant predictor of diastolic blood pressure reduction, along with gender. Furthermore, in the model developed for weighted SD diastolic, it emerged as a predictor along with BMI. High insulin levels have been shown to cause an increase in blood pressure due to sympathetic nervous system activation and sodium accumulation in the kidneys [36,37]. Since C-peptide is an indicator of insulin secretion, it can also be used to detect similar relationships. In our study, a decrease in C-peptide levels (β = 0.246, *p* = 0.013) suggests a positive effect on blood pressure, independent of glycemic control. As a result, our study revealed that C-peptide can be a blood pressure predictor in a non-diabetic population undergoing bariatric surgery.

### 4.5. Improvement in Autonomic Functions

Our study showed that HRV parameters have different predictors. While most time-domain HRV parameters (pNN30, pNN50, RMSSD) were predicted by HbA1c or HOMA-IR (β = 0.240–0.281, *p* < 0.05), no significant predictor emerged for frequency-domain HRV parameters. We also found a significant decrease in heart rate after LSG was predicted by BMI rather than metabolic factors (β = 0.340, *p* = 0.002). These findings suggest that time-domain HRV indices may be more closely associated with metabolic changes, whereas heart rate reduction appeared related to BMI reduction.

In the study by Wu et al., an independent association was found between the decrease in HOMA-IR index and the increases in RMSSD and HF bands on day 180 postoperatively; our findings are consistent with theirs [38]. Perugini et al. also demonstrated that HRV improvement was inversely proportional to changes in fasting insulin concentrations independently of weight loss [30]. A meta-analysis in patients with type 2 DM revealed that HbA1c reduction was an independent predictor for improvement in SDNN, RMSSD, and LF/HF [39]. This meta-analysis also showed that reductions in systolic and diastolic BP are independent predictors for RMSSD [39]. Similarly, in our study, we detected significant relationships between reductions in HbA1c, glucose, and HOMA-IR parameters and improvements in HRV parameters, while also identifying statistically significant correlations between improvements in blood pressure parameters and HRV parameters. In the study by McGee et al. investigating the effect of weight loss on cardiac autonomic function in obese patients, regression analysis showed fasting insulin levels as an independent predictor for RMSSD and HF [40].

HbA1c reduction (β = 0.377, *p* = 0.004) and male sex (β = −0.261, *p* = 0.041) were associated with changes in TS (R^2^ = 20.8%), although the small male subgroup (n = 24, 30.8%) limits the stability of this gender-based finding. The SDANN model showed lower explanatory power (R^2^ = 6.6%). This suggests that baroreflex recovery is more closely related to glycemic control. In the literature, a study in class III obese young patients performed HRT analysis in 32 patients and demonstrated that TS values and abnormal HRT prevalence were significantly different in the severely obese group compared with the control group [41]. However, in the prospective study by the same investigators in patients who underwent LSG, no significant difference was found in HRT parameters between preoperative and postoperative 12–18 months [19]. A significant limitation of this study was the inclusion of only 10 patients eligible for HRT analysis. HRT analysis performed on 53 patients showed significant improvements in both components. TO became more negative from −0.54 ± 1.73 to −2.53 ± 1.97 (*p* < 0.001), reflecting improved cardiac autonomic control, while TS rose from 5.98 ± 3.49 ms/RR to 9.87 ± 4.28 ms/RR (*p* < 0.05), indicating enhanced baroreflex sensitivity after surgery. Compared to the study by Bienias et al., both TO and TS showed significant improvement in our cohort of 53 patients eligible for HRT analysis [19].

Another important finding of our study is that male sex emerged as a predictor factor for both diastolic blood pressure reduction (β = −0.360, *p* < 0.001) and turbulence slope improvement (β = −0.261, *p* = 0.041). However, due to the gender imbalance in our study (women 69%, n = 54; men 31%, n = 24), this finding should be approached with caution. This finding needs to be confirmed in larger and gender-balanced cohorts before drawing definitive conclusions about gender-specific cardiovascular outcomes after LSG.

### 4.6. Clinical Significance of Observed Changes

While our findings are statistically significant, they warrant cautious interpretation of clinical outcomes. Improvements in heart rate variability (HRV) parameters—an increase in SDNN from 95 ms to 113 ms and RMSSD from 27 ms to 37 ms—reached levels previously associated with lower cardiovascular risk in older or high-risk populations (SDNN > 100 ms, RMSSD > 30 ms). However, it is unclear whether these improvements translate into a significant risk reduction in our relatively young (mean age 36) cohort with undiagnosed cardiovascular disease, and long-term outcome studies are required.

Regarding HRT, threshold values of TO < 0% and TS > 2.5 ms/RR have been established to indicate preserved baroreflex sensitivity [18]. In our cohort, postoperative values were within the accepted normal range (TO < 0%, TS > 2.5 ms/RR). While there are no universal threshold values for BPV, lower standard deviation values are associated with better cardiovascular outcomes, and the reductions we observed in all BPV indices may be clinically significant.

Similarly, the reduction in the proportion of non-dipper from 67.9% to 53.8% represents a modest absolute improvement, with more than half of the patients remaining postoperatively non-dipper. In a subgroup of patients, the clinical benefit of restoring partial nocturnal blood pressure reduction is unclear, particularly without assessment of target organ parameters such as left ventricular mass or albuminuria. In contrast, significant reductions in BPL (27% reduction in systolic BPL, 30% reduction in diastolic BPL) and BPV may be more clinically significant. These parameters may indicate a more favorable hemodynamic profile associated with reduced cardiovascular risk.

### 4.7. Limitations

The present study has important limitations. The six-month follow-up period limits assessment of long-term cardiovascular and autonomic adaptation, which may develop differently for weight-related blood pressure parameters and metabolism-related autonomic function. In addition, weight regain or metabolic adaptation beyond the early postoperative period may diminish these effects; thus, our findings should be considered preliminary and confirmed with longer-term follow-up and definitive clinical outcomes. This single-center study includes a relatively small cohort of the Turkish population; this may restrict external validity.

The lack of a control group precludes definitive causal conclusions, as regression to the mean, lifestyle changes, postoperative care, or temporal effects cannot be fully excluded. Accordingly, the observed improvements should be interpreted as associations rather than evidence of causality. A substantial exclusion rate (84 of 162 patients) and the exclusion of patients requiring long-term antihypertensive therapy may have introduced selection bias. The exclusion of patients requiring antihypertensive treatment at 3 months postoperatively (n = 14 from the original cohort) represents an important limitation. These patients likely had more severe or treatment-resistant hypertension and may have exhibited different autonomic dysfunction patterns. This exclusion was necessary to avoid the confounding effects of antihypertensive medications on blood pressure and HRV measurements. However, by excluding this higher-risk subgroup, our results may overestimate the magnitude of autonomic improvements achievable with LSG in the broader population of severely obese patients. The true effect size in an unselected bariatric population likely falls between our observed improvements and the potentially more modest changes in patients requiring ongoing antihypertensive therapy. Future prospective studies should include patients on stable antihypertensive regimens with stratified analysis to fully characterize the spectrum of autonomic responses across different cardiovascular risk profiles. As a consequence, our findings apply not to all bariatric surgery candidates, but to a selected subgroup of severely obese patients who successfully completed LSG without requiring long-term antihypertensive therapy. Additionally, the exclusion of patients from the HRT analysis due to inadequate ventricular premature beats may further limit the generalizability of autonomic findings.

The low sample size relative to the number of relationships tested (n = 78; HRT n = 53) limits the stability of regression models and raises concerns regarding model stability. The use of stepwise regression carries a risk of overfitting, particularly given the sample size relative to the number of candidate predictors. In addition, the modest adjusted R^2^ values observed in several models indicate that only a limited proportion of outcome variance was explained. Accordingly, these regression findings should be considered exploratory and require validation in independent cohorts. The modest adjusted R^2^ values suggest substantial unexplained variance, and Type I error inflation cannot be excluded. In addition, unmeasured factors, including gastrointestinal hormones, vasoactive mediators, and other biological mechanisms known to influence blood pressure and autonomic function after LSG, may have contributed to the observed effects. The predominance of female participants limits the interpretation of sex-related findings and warrants confirmation in larger, gender-balanced cohorts.

Moreover, we did not measure dietary sodium intake, physical activity levels, or assess sleep apnea via polysomnography. Postoperative weight loss is typically accompanied by changes in diet, activity, and sleep, each of which may independently influence blood pressure and autonomic function. Without these measurements, we cannot separate the effects of weight loss from lifestyle changes. Improvements likely reflect combined effects rather than weight loss alone. Given the numerous statistical comparisons performed across BP, BPV, HRV, HRT, correlation, and regression analyses, the possibility of type I error must be considered. Since this study was designed as an exploratory, hypothesis-generating investigation, formal correction for multiple testing was not applied. As a result, observed associations should be interpreted with caution and validated in larger, adequately powered studies.

## 5. Conclusions

In this prospective analysis, laparoscopic sleeve gastrectomy was associated with significant improvements in 24 h blood pressure profile, blood pressure variability, and cardiac autonomic function within six months in severely obese individuals without diagnosed hypertension or diabetes.

Weight reduction was primarily linked to improvements in ambulatory blood pressure and variability indices, whereas improvements in glycemic markers—particularly HbA1c and HOMA-IR—were more closely associated with recovery of autonomic parameters and baroreflex sensitivity. These findings suggest that early cardiovascular adaptation after LSG may involve partially distinct weight-related and metabolic pathways.

The high prevalence of masked hypertension identified preoperatively further emphasizes the value of ambulatory blood pressure monitoring in severely obese patients. Taken together, our results indicate that LSG is associated with early multidimensional cardiovascular remodeling extending beyond weight reduction alone.

## Figures and Tables

**Table 1:** Baseline Demographic, Anthropometric and Echocardiographic Characteristics.

Variable	Mean ± SD	Median (IQR)	Range
Demographics			
Age (years)	36.04 ± 11.34	-	18–62
Female, n (%)	54 (69.2%)	-	-
Height (cm)	164.41 ± 9.25	165.00 (11)	142–190
Smoking, n (%)	28 (35.9%)	-	-
Hyperlipidemia, n (%)	33 (42.3%)	-	-
Anthropometric Parameters			
Preoperative weight (kg)	127.97 ± 24.57	123.00 (26)	87–210
6-month weight (kg)	84.94 ± 15.57	81.00 (15)	62–135
Total weight loss (kg)	43.04 ± 13.65	*p* < 0.001 ‡	18–100
Preoperative BMI (kg/m^2^)	47.21 ± 7.46	45.19 (9.22)	39.86–82.03
6-month BMI (kg/m^2^)	31.45 ± 5.28	30.04 (5.92)	19.39–52.73
BMI reduction (kg/m^2^)	15.75 ± 4.05	*p* < 0.001 ‡	7.12–34.60
Echocardiographic Parameters			
LVDD (cm)	4.74 ± 0.42	4.70 (0.60)	4.00–5.70
LVDS (cm)	3.14 ± 0.47	3.00 (0.60)	2.30–4.80
LVEF (%)	64.49 ± 2.97	65.00 (0)	55–70
LA diameter (cm)	3.90 ± 0.46	3.80 (0.52)	2.90–5.50
IVS thickness (cm)	1.04 ± 0.11	1.00 (0.10)	0.70–1.40
PW thickness (cm)	0.94 ± 0.10	0.90 (0.10)	0.70–1.20

Distribution assessed by Shapiro—Wilk test (*p* > 0.05 indicates normal distribution), ‡ Wilcoxon signed-rank test. BMI: body mass index; IQR: interquartile range; IVS: interventricular septum; LA: left atrium; LVDD: left ventricular end-diastolic diameter; LVDS: left ventricular end-systolic diameter; LVEF: left ventricular ejection fraction; PW: posterior wall; SD: standard deviation.

**Table 2:** Comparison of Laboratory Parameters Before and 6 Months After Sleeve Gastrectomy.

Parameter	Preoperative	6-Month	Change	*p*-Value
Hematologic Parameters				
WBC (×10^3^/µL)	9.49 ± 2.34	7.21 ± 1.86	−2.28 ± 1.96	**<0.001 †**
Hemoglobin (g/dL)	13.01 ± 1.92	13.47 ± 1.38	+0.46 ± 1.37	**0.004 †**
Platelet (×10^3^/µL)	300.35 ± 74.15	278.38 ± 67.08	−21.96 ± 50.46	**<0.001 †**
Renal Function Parameters				
BUN (mg/dL)	10.1 (8.4–12.2)	10.8 (9.1–13.5)	+0.47	0.102 ‡
Creatinine (mg/dL)	0.64 (0.57–0.77)	0.68 (0.60–0.77)	−0.01	0.845 ‡
eGFR (mL/min/1.73 m^2^)	115.9 (106.2–125.2)	115.7 (108.7–121.3)	+0.37	0.530 ‡
Potassium (mEq/L)	4.58 ± 0.44	4.58 ± 0.37	0.00 ± 0.45	1.000 †
Calcium (mg/dL)	9.17 ± 0.50	9.25 ± 0.39	+0.08 ± 0.47	0.124 †
Metabolic Parameters				
Fasting glucose (mg/dL)	97.5 (91.0–109.2)	86.0 (81.8–92.0)	−13.92	**<0.001 ‡**
HbA1c (%)	5.5 (5.2–6.0)	5.1 (4.9–5.3)	−0.49	**<0.001 ‡**
Fasting insulin (µIU/mL)	22.5 (14.1–31.0)	8.6 (5.9–11.8)	−15.51	**<0.001 ‡**
C-peptide (ng/mL)	3.7 (3.1–4.8)	2.4 (1.9–3.1)	−1.54	**<0.001 ‡**
HOMA-IR	5.6 (3.3–7.7)	1.8 (1.2–2.7)	−4.33	**<0.001 ‡**
Lipid Parameters				
Total cholesterol (mg/dL)	203.62 ± 39.45	184.50 ± 30.47	−19.12 ± 22.54	**<0.001 †**
LDL-cholesterol (mg/dL)	128.82 ± 33.35	115.89 ± 26.63	−12.93 ± 14.15	**<0.001 †**
HDL-cholesterol (mg/dL)	44.85 ± 10.66	46.26 ± 10.67	+1.40 ± 5.65	**0.031 †**
Triglyceride (mg/dL)	146.21 ± 50.59	126.68 ± 39.31	−19.53 ± 35.43	**<0.001 †**

Data presented as mean ± standard deviation for normally distributed variables or median (Q1–Q3) for non-normally distributed variables. † Paired *t*-test, ‡ Wilcoxon signed-rank test. Bold values indicate statistical significance (*p* < 0.05). BUN: blood urea nitrogen; eGFR: estimated glomerular filtration rate; HbA1c: glycated hemoglobin; HDL: high-density lipoprotein; LDL: low-density lipoprotein; Q1: first quartile (25th percentile); Q3: third quartile (75th percentile); WBC: white blood cell count.

**Table 3:** Comparison of 24-Hour Ambulatory Blood Pressure Parameters Before and 6 Months After Sleeve Gastrectomy.

Parameter	Preoperative	6-Month	Change	*p*-Value
24-Hour Blood Pressure				
24 h SBP (mmHg)	139 (133–144)	125 (119–130)	−14.45	**<0.001**
24 h DBP (mmHg)	81 (78–85)	71 (65–76)	−10.77	**<0.001**
24 h MAP (mmHg)	103 (98–106)	89 (84–93)	−11.99	**<0.001**
Daytime Blood Pressure				
Daytime SBP (mmHg)	142 (138–145)	128 (121–135)	−14.39	**<0.001**
Daytime DBP (mmHg)	86 (81–91)	73 (69–77)	−12.45	**<0.001**
Daytime MAP (mmHg)	105 (100–110)	91 (87–95)	−13.09	**<0.001**
Nighttime Blood Pressure				
Nighttime SBP (mmHg)	131 (124–138)	116 (106–123)	−16.22	**<0.001**
Nighttime DBP (mmHg)	75 (69–80)	64 (57–67)	−12.11	**<0.001**
Nighttime MAP (mmHg)	94 (88–99)	80 (74–86)	−13.49	**<0.001**
Blood Pressure Load				
24 h SBP load (%)	40 (31–43)	10 (7–15)	−27.09	<0.001
24 h DBP load (%)	40 (24–50)	5 (0–15)	−30.19	<0.001
Blood Pressure Variability				
24 h SBP SD (mmHg)	15.5 (12.1–18.1)	10.3 (7.8–12.6)	−3.75	**<0.001**
24 h DBP SD (mmHg)	13.9 (11.1–15.6)	9.1 (6.7–11.1)	−4.01	**<0.001**
SBP coefficient of variation (%)	13.1 (10.7–15.3)	9.9 (8.8–12.7)	−3.18	**<0.001**
DBP coefficient of variation (%)	20.4 (16.8–26.2)	14.3 (12.0–17.1)	−6.86	**<0.001**
Weighted SBP SD (mmHg)	13.2 (10.7–17.2)	11.2 (9.0–14.6)	−2.14	**<0.001**
Weighted DBP SD (mmHg)	13.6 (10.9–15.4)	8.6 (6.8–10.4)	−4.65	**<0.001**
Dipping Status				
Systolic dipping (%)	8.5 (5.9–10.6)	9.5 (5.4–13.0)	+0.94	0.323
Diastolic dipping (%)	12.8 (8.6–16.0)	13.2 (9.1–19.6)	+1.78	0.102
Non-dippers, n (%)	53 (67.9%)	42 (53.8%)	−14.1%	**0.013**

Data presented as median (Q1–Q3) unless otherwise indicated. All comparisons performed using Wilcoxon signed-rank test. Bold values indicate statistical significance (*p* < 0.05). DBP: diastolic blood pressure; MAP: mean arterial pressure; Q1: first quartile (25th percentile); Q3: third quartile (75th percentile); SBP: systolic blood pressure; SD: standard deviation.

**Table 4:** Baseline Characteristics and Post-LSG Changes in Masked Hypertension vs. Normal/High-Normal Blood Pressure Groups.

Parameter	Masked HT n = 34 (43.5%)	Normal/High-Normal n = 44 (56.5%)	*p*-Value
Baseline Data			
Age (years)	31.5 (25.0–43.3)	38.5 (28.0–45.8)	0.083
BMI (kg/m^2^)	48.1 (43.8–54.4)	43.8 (40.5–48.0)	**0.001**
24 h SBP (mmHg)	140.5 (137.8–145.3)	135.0 (131.0–143.0)	**0.001**
24 h DBP (mmHg)	85.0 (78.0–88.0)	81.0 (78.3–82.0)	**0.005**
SBP Load (%)	40.0 (35.0–47.3)	36.5 (30.0–40.8)	**0.029**
DBP Load (%)	42.5 (30.8–60.0)	38.5 (20.0–50.0)	**0.050**
Heart Rate (bpm)	90.0 (84.5–94.0)	84.5 (78.5–89.0)	**0.024**
SDNN (ms)	43.6 (33.3–53.5)	41.6 (34.0–53.8)	0.604
SDANN (ms)	96.2 (76.8–117.1)	88.5 (71.7–112.5)	0.468
TO (%) * (n = 26 vs. 27)	−0.88 (−1.79–0.95)	−1.00 (−1.64–1.34)	0.722
TS (ms/RR) * (n = 26 vs. 27)	4.58 (3.09–7.33)	5.50 (3.00–10.15)	0.482
RMSSD (ms)	27.8 (19.0–35.4)	24.5 (19.6–36.0)	0.755
Glucose (mg/dL)	103.5 (92.0–109.9)	97.0 (90.3–108.8)	0.459
Insulin (µIU/mL)	27.6 (21.0–40.0)	17.9 (12.6–25.3)	**<0.001**
C-peptide (ng/mL)	4.51 (3.63–5.65)	3.35 (2.96–4.12)	**0.001**
HbA1c (%)	5.85 (5.20–6.20)	5.40 (5.23–5.70)	0.070
HOMA-IR	7.06 (4.87–11.02)	4.20 (2.78–6.69)	**<0.001**
Post-LSG Changes (Δ Values)			
Δ BMI (kg/m^2^) †	−16.0 (−19.1 to −14.6)	−14.5 (−15.9 to −12.6)	**<0.001**
Δ 24 h SBP (mmHg) *	−16.94 ± 5.56	−15.02 ± 5.89	0.123
Δ 24 h DBP (mmHg) *	−13.29 ± 4.69	−12.14 ± 6.08	0.286
Δ SDANN (ms) *	+13.37 ± 22.21	+22.81 ± 28.46	0.225
Δ RMSSD (ms) †	+8.4 (+5.1 to +11.6)	+8.8 (+5.4 to +13.1)	0.450
Δ pNN50 (%) †	+4.0 (+2.0 to +5.9)	+4.4 (+2.9 to +6.8)	0.285
Δ Glucose (mg/dL) *	−15.55 ± 15.18	−12.66 ± 12.08	0.173
Δ Insulin (µIU/mL) †	−17.2 (−27.5 to −8.6)	−9.4 (−15.0 to −5.5)	**0.003**
Δ C-peptide (ng/mL) †	−1.59 (−2.78 to −0.97)	−1.16 (−1.46 to −0.57)	**0.013**
Δ HbA1c (%) *	−0.59 ± 0.48	−0.41 ± 0.31	0.092
Δ TO (%) * (n = 26 vs. 27)	−1.54 ± 1.95	−2.42 ± 1.36	**0.027**
Δ TS (ms/RR) * (n = 26 vs. 27)	+3.35 ± 1.68	+4.42 ± 2.58	0.165

Baseline data presented as median (Q1–Q3). Post-LSG changes presented as * mean ± SD for normally distributed variables or † median (Q1–Q3) for non-normally distributed variables. Baseline comparisons: Mann–Whitney U test. Post-LSG changes: * Independent *t*-test for normally distributed variables; † Mann–Whitney U test for non-normally distributed variables. Bold values indicate statistical significance (*p* < 0.05). BMI: body mass index; DBP: diastolic blood pressure; HbA1c: hemoglobin A1c; HOMA-IR: homeostatic model assessment of insulin resistance; HT: hypertension; LSG: laparoscopic sleeve gastrectomy; pNN50: percentage of successive NN intervals differing by >50 ms; Q1: first quartile (25th percentile); Q3: third quartile (75th percentile); RMSSD: root mean square of successive differences; SBP: systolic blood pressure; SDANN: standard deviation of averaged NN intervals; SDNN: standard deviation of NN intervals; TO: turbulence onset; TS: turbulence slope.

**Table 5:** Comparison of Heart Rate Variability, Heart Rate Turbulence, and Arrhythmic Parameters Before and 6 Months After Sleeve Gastrectomy.

Parameter	Preoperative	6-Month	Change	*p*-Value
Heart Rate				
Mean heart rate (bpm)	87 (81–92)	77 (71–81)	−9.98	**<0.001**
Total QRS complexes	120,647 (112,818–131,403)	108,374 (103,171–115,341)	−11,981	**<0.001**
Time-Domain HRV Parameters				
SDNN (ms)	42.7 (33.9–53.3)	57.5 (46.6–66.2)	+14.26	**<0.001**
SDANN (ms)	92.4 (73.5–114.2)	109.9 (89.5–144.7)	+18.69	**<0.001**
RMSSD (ms)	26.7 (19.5–35.6)	36.5 (27.7–44.7)	+9.74	**<0.001**
pNN50 (%)	3.4 (1.5–10.0)	9.5 (5.9–14.2)	+4.87	**<0.001**
pNN30 (%)	13.2 (6.1–19.3)	21.3 (14.2–28.8)	+8.08	**<0.001**
Variability index	2.1 (1.7–2.8)	3.1 (2.6–3.7)	+0.82	**<0.001**
Frequency-Domain HRV Parameters				
Total power (ms^2^)	1893 (1338–2702)	3269 (2215–3867)	+1082	**<0.001**
VLF power (ms^2^)	1224 (959–1709)	2037 (1580–2730)	+810	**<0.001**
LF power (ms^2^)	524 (349–759)	849 (541–1160)	+348	**<0.001**
HF power (ms^2^)	159 (102–395)	351 (236–579)	+164	**<0.001**
LF normalized units	61.3 (48.8–65.6)	56.0 (45.9–63.1)	−4.80	**<0.001**
HF normalized units	22.8 (17.4–30.5)	25.7 (22.0–34.4)	+4.31	**<0.001**
LF/HF ratio	2.7 (1.7–3.6)	2.0 (1.4–2.7)	−0.63	**<0.001**
Heart Rate Turbulence (N = 53)				
Turbulence onset (%)	−0.92 (−1.71 to 1.29)	−2.49 (−3.72 to −0.55)	−1.99	**<0.001**
Turbulence slope (ms/RR)	5.0 (3.0–7.9)	9.0 (6.7–12.8)	+3.89	**<0.001**
Arrhythmic Events				
Total APCs (n)	26 (9–164)	56 (17–328)	−286	**<0.001**
SV runs (n)	0 (0–6)	2 (0–8)	+1.33	0.753
Total VPCs (n)	122 (25–543)	224 (58–965)	+109	0.439

Data presented as median (Q1–Q3). All comparisons performed using Wilcoxon signed-rank test. Bold values indicate statistical significance (*p* < 0.05). APC: atrial premature complex; HF: high frequency; HRV: heart rate variability; LF: low frequency; pNN30: percentage of successive NN intervals differing by >30 ms; pNN50: percentage of successive NN intervals differing by >50 ms; Q1: first quartile (25th percentile); Q3: third quartile (75th percentile); RMSSD: root mean square of successive differences; SDANN: standard deviation of averaged NN intervals; SDNN: standard deviation of NN intervals; SV: supraventricular; VLF: very low frequency; VPC: ventricular premature complex.

**Table 6:** Correlation Analysis Between Weight Loss and Cardiovascular/Autonomic Parameters.

Parameter Correlation	r Value	*p*-Value
Weight Loss Correlations		
Total weight loss vs. 24 h SBP reduction	**0.258**	**0.022**
Total weight loss vs. 24 h DBP reduction	**0.350**	**0.002**
Total weight loss vs. SDANN improvement	**0.278**	**0.014**
Total weight loss vs. pNN50 improvement	**0.268**	**0.018**
Total weight loss vs. HF power improvement	**0.249**	**0.028**
Total weight loss vs. TO improvement (n = 53)	**−0.310**	**0.024**
Total weight loss vs. TS improvement (n = 53)	**0.309**	**0.024**
BMI Reduction Correlations		
BMI reduction vs. Weighted SD diastolic reduction	**0.357**	**0.001**
BMI reduction vs. MAP reduction	**0.333**	**0.003**
BMI reduction vs. TS improvement (n = 53)	**0.286**	**0.038**
BMI reduction vs. TO improvement (n = 53)	**−0.274**	**0.047**
BMI reduction vs. LF/HF ratio reduction	**−0.228**	**0.045**
Metabolic Parameter Correlations		
Insulin reduction vs. weight loss	**0.435**	**<0.001**
HbA1c reduction vs. TS improvement (n = 53)	**0.436**	**0.001**
Insulin reduction vs. pNN50 improvement	**0.274**	**0.015**
Insulin reduction vs. C-peptide reduction	**0.718**	**<0.001**
HOMA-IR Correlations		
HOMA-IR ↔ Total Weight Loss	r = 0.454	*p* < 0.001
HOMA-IR ↔ DBP Reduction	r = 0.356	*p* = 0.001
HOMA-IR ↔ MAP Reduction	r = 0.351	*p* = 0.002
HOMA-IR ↔ Weighted SD Diastolic	r = 0.320	*p* = 0.004
HOMA-IR ↔ pNN50	r = 0.257	*p* = 0.023
BP Variability vs. HRV Correlations		
CV diastolic reduction vs. Variability Index improvement	**0.358**	**0.001**
CV diastolic reduction vs. LF/HF ratio reduction	**−0.276**	**0.014**
HF power improvement vs. LF/HF ratio reduction	**−0.335**	**0.003**

Data analyzed using Spearman’s rank correlation coefficient. Bold values indicate statistical significance (*p* < 0.05). Interpretation: Weak (0.2–0.39), Moderate (0.4–0.59), Strong (0.6–0.79), Very Strong (≥0.8). CV: coefficient of variation; DBP: diastolic blood pressure; HF: high frequency; HRV: heart rate variability; LF: low frequency; MAP: mean arterial pressure; pNN50: percentage of successive NN intervals differing by >50 ms; RMSSD: root mean square of successive differences; SBP: systolic blood pressure; SD: standard deviation; SDANN: standard deviation of averaged NN intervals; TO: turbulence onset; TS: turbulence slope.

**Table 7:** Summary of Multivariate Regression Models—Independent Predictors of Cardiovascular and Autonomic Outcomes.

Outcome Variable	Independent Predictors (β, *p*-Value)	95% CI	R^2^	F	*p*-Value
Autonomic Function Outcomes					
Heart Rate reduction (bpm)	BMI reduction (β = 0.340, *p* = 0.002)	0.16–0.71	0.116	9.928	**0.002**
SDANN improvement (ms)	Glucose reduction (β = 0.279, *p* = 0.013)	0.12–0.97	0.066	6.415	**0.013**
pNN50 improvement (%)	HOMA-IR reduction (β = 0.240, *p* = 0.034)	0.02–0.56	0.058	4.665	**0.034**
pNN30 improvement (%)	HbA1c reduction (β = 0.281, *p* = 0.013)	0.98–7.93	0.079	6.518	**0.013**
RMSSD improvement (ms)	HbA1c reduction (β = 0.236, *p* = 0.038)	0.28–9.48	0.056	4.471	**0.038**
TS improvement (ms/RR)	HbA1c reduction (β = 0.377, *p* = 0.004) Male gender (β = −0.261, *p* = 0.041)	0.67–3.33 −2.36/−0.05	0.208	7.826	**0.001**
Blood Pressure Outcomes					
24 h DBP reduction (mmHg)	Male gender (β = −0.360, *p* < 0.001) Glucose reduction (β = 0.309, *p* = 0.002) C-peptide reduction (β = 0.246, *p* = 0.013)	−6.56/−1.99 0.05–0.21 0.24–1.96	0.297	11.833	**<0.001**
24 h DBP Alternative Model	Male gender (β = −0.339, *p* = 0.001) HOMA-IR reduction (β = 0.321 *p* = 0.002)	−6.41/−1.63 0.18–0.78	0.252	12.602	**<0.001**
Blood Pressure Load Outcomes					
SBP Load reduction (%)	Glucose reduction (β = 0.293, *p* = 0.009)	0.12–0.84	0.074	7.158	**0.009**
DBP Load reduction (%)	BMI reduction (β = 0.244, *p* = 0.031)	0.12–2.52	0.047	4.808	**0.031**
Blood Pressure Variability Outcomes					
SBP SD reduction (mmHg)	BMI reduction (β = 0.276, *p* = 0.014)	0.08–0.67	0.064	6.273	**0.014**
DBP SD reduction (mmHg)	BMI reduction (β = 0.289, *p* = 0.010)	0.07–0.50	0.071	6.928	**0.010**
Weighted SD Systolic reduction	BMI reduction (β = 0.338, *p* = 0.002)	0.10–0.43	0.103	9.821	**0.002**
Weighted SD Diastolic reduction	BMI reduction (β = 0.413, *p* < 0.001)	0.17–0.51	0.160	15.641	**<0.001**
Weighted SD Diastolic—Enhanced	BMI reduction (β = 0.307, *p* = 0.010) HOMA-IR reduction (β = 0.231, *p* = 0.048)	0.06–0.44 0.00–0.41	0.213	10.145	**<0.001**
CV Systolic reduction (%)	BMI reduction (β = 0.241, *p* = 0.033)	0.02–0.44	0.046	4.691	**0.033**
CV Diastolic reduction (%)	BMI reduction (β = 0.225, *p* = 0.047)	0.00–0.54	0.038	4.062	**0.047**

All models used stepwise linear regression (n = 78 for all models except TS where n = 53). β represents standardized coefficients. 95% CI = 95% confidence interval for unstandardized B coefficients. Variance inflation factor (VIF) was <2.5 for all predictors (range 1.02–1.27), indicating no multicollinearity. Bold *p*-values indicate statistical significance (*p* < 0.05). R^2^ = Adjusted R^2^. Gender-based predictors should be interpreted with caution given the gender imbalance (males n = 24, 30.8%; females n = 54, 69.2%). Abbreviations: BMI, body mass index; CI, confidence interval; CV, coefficient of variation; DBP, diastolic blood pressure; HbA1c, hemoglobin A1c; HOMA-IR, homeostatic model assessment of insulin resistance; SBP, systolic blood pressure; SD, standard deviation; SDANN, standard deviation of averaged NN intervals; TS, turbulence slope; VIF, variance inflation factor.

## Data Availability

The data used in this study and included in the analyses are available from the corresponding author upon reasonable request.
